# Supplementary material for: Mechanisms governing the pioneering and redistribution capabilities of the non-classical pioneer PU.1
Source: Nat Commun. 2020 Jan 21;11:402. doi: 10.1038/s41467-019-13960-2 (PMC6972792; doi:10.1038/s41467-019-13960-2)
Supplement: Supplementary file 7 — Source data [file 41467_2019_13960_MOESM7_ESM.zip › Source_Data/FigureS2/FigureS2B_MotifScanOutput/KmeansCluster2/homerResults/motif35.similar.html]

motif35

## Information for motif35

C
T
A
G
C
G
T
A
A
C
T
G
C
T
A
G
A
C
G
T
A
C
T
G
C
G
T
A
A
G
C
T
A
G
C
T
A
C
T
G
  
Reverse Opposite:  

A
G
T
C
C
T
G
A
C
T
G
A
A
C
G
T
A
G
T
C
C
G
T
A
A
G
T
C
A
G
T
C
A
C
G
T
A
G
T
C
  

|  |  |
| --- | --- |
| p-value: | 1e-28 |
| log p-value: | -6.657e+01 |
| Information Content per bp: | 1.901 |
| Number of Target Sequences with motif | 86.0 |
| Percentage of Target Sequences with motif | 1.71% |
| Number of Background Sequences with motif | 121.3 |
| Percentage of Background Sequences with motif | 0.30% |
| Average Position of motif in Targets | 107.6 +/- 78.8bp |
| Average Position of motif in Background | 90.1 +/- 67.0bp |
| Strand Bias (log2 ratio + to - strand density) | -0.2 |
| Multiplicity (# of sites on avg that occur together) | 1.00 |
| Motif File: | file (matrix) reverse opposite |

### Similar de novo motifs found

|  |  |  |  |  |  |  |  |
| --- | --- | --- | --- | --- | --- | --- | --- |
| Rank | Match Score | Redundant Motif | P-value | log P-value | % of Targets | % of Background | Motif file |
| 1 | 0.909 | G T C A A C T G A C T G A C G T A C T G C G T A A C G T A G C T A C T G | 1e-19 | -45.912954 | 1.19% | 0.22% | motif file (matrix) |
| 2 | 0.825 | A C T G C T A G A G C T A T C G C G T A A C G T A G T C A C T G | 1e-15 | -36.325762 | 1.81% | 0.60% | motif file (matrix) |
